# Rural influences on the social network dynamics of district nursing services: A qualitative meta‐synthesis

**DOI:** 10.1002/hsr2.336

**Published:** 2021-08-17

**Authors:** Jack Gillham, Ivaylo Vassilev, Rebecca Band

**Affiliations:** ^1^ School of Health Sciences University of Southampton Southampton England

**Keywords:** community nurse, district nurse, nursing, rural, rurality, social networks, social support

## Abstract

**Background and Aims:**

As demands on healthcare services grow, fiscal restrictions place increased emphasis on services outside of traditional healthcare settings. Previous research into long‐term‐conditions suggests that social network members (including weaker ties such as acquaintances, community groups, and healthcare professionals) play a key role in illness management. There is limited knowledge about the engagement of social networks in supporting people who are receiving medical interventions at home. This qualitative metasynthesis explores the work and the interactions between district nurses (DN) and informal network members supporting people who are receiving medical interventions at home and living in rural areas.

**Methods:**

A search was undertaken on CINAHL, Medline, and PsychINFO for qualitative research articles from 2009 to 2019. Studies that examined DN in rural locations and/or social network support in rural locations were eligible. Fourteen articles were selected.

**Results:**

Thematic analysis of results and discussion data from the studies resulted in four themes being developed: the development of both transactional and friend‐like nurse‐patient ties in rural localities, engagement of the wider network in the delivery of good care, blurring of professional boundaries in close community relationships, and issues accessing and navigating formal and informal support in the context of diminishing resources in rural areas.

These findings suggest that DNs in rural localities work beyond professional specialties and experience to provide emotional support, help with daily tasks, and build links to communities. There was also evidence that nurses embedded within rural localities developed friend‐like relationships with patients, and negotiated with existing support networks and communities to find support for the patient.

**Conclusions:**

Findings indicated that developing strong links with patients and members of their networks does not automatically translate into positive outcomes for patients, and can be unsustainable, burdensome, and disruptive. DNs developing weak ties with patients and building awareness of the structure of individual networks and local sources of support offers avenues for sustainable and tailored community‐based nursing support.

## 1 INTRODUCTION

Aging populations, driven by falling infant death rates, longer life expectancies, and increased availability of medical and pharmaceutical interventions^1^ – coupled with uncertain financial climates, rising populations, and increased co‐morbidities has led to escalating costs, a high demand on hospital beds,^2^ and profound changes in how healthcare is delivered.^1^ Rising healthcare need and a slowdown in funding have led to National Health Service (NHS) debt rising to £13.4 billion in 2020.^3^

As a way of addressing this gap in funding, more patients are being treated in community settings for increasingly complex conditions to reduce the costs associated with inpatient admission.^4^, ^5^ Understanding the consequences of this shift in healthcare delivery style is set to increase in pertinence as wider policy moves health and social care closer to home and community settings.^6^ For example, the implementation of the “Long Term Plan” in the NHS in the UK (2019) emphasizes community healthcare by stating an annual primary care budget increase of £4.5 bn. These policies aim to increase service capacity and establish new provisions (such as “hospital at home” [HAH]) services, online consultation services, increased general practitioner (GP) training, and “same day emergency care” units) that overcome barriers currently preventing some health conditions being treated at home by, for example, guaranteeing online tele‐health consultations, and direct referrals to community services that means unnecessary visits to hospital can be avoided.^2^

### 1.1 Social networks and self‐management

The role of social networks in supporting self‐management has most extensively been researched in the context of long‐term condition management.^7^, ^8^ This research has focused on how the structure of people's networks and the types and range of relationships shape the way in which people access different types of support with their health, practical, and emotional needs.^9^ Such studies have found that people who have access to diverse types of relationships, including both “strong,” intimate ties (eg, family members, close friends) and “weak,” more distant ties (eg, healthcare professionals [HCPs], acquaintances, and community group members) are most successful in managing their long term conditions (LTC) and receiving acceptable health, emotional, and practical support.^10^, ^11^, ^12^ This might be in part due to such networks being able to share the burden of illness work, sustain valued relationships, and have better access to relevant knowledge, skills, and experience.^13^ Consequently, access to social network support that is acceptable to people may facilitate improved use of medications, healthier lifestyles (eg, smoking cessation or healthy eating practices),^14^, ^15^ improved experiences of poor health (eg, by managing adjustment; sharing the burden of health, emotional and practical work; and advocating/liaising with HCPs),^16^ and improved physical and mental wellbeing.^10^

The increased focus on the community provision of healthcare is likely to result in a widening of the types of conditions and issues that require patients to self‐manage. Beyond LTCs, this is likely to include potentially complex and acute conditions, both of which will require further research into the specific roles of social networks in these different contexts.

### 1.2 The healthcare professional's role in self‐management support

Previous research has suggested that in a community context, HCPs may facilitate self‐management by offering not only health work, but emotional and practical work too.^9^, ^13^ However, the quality and acceptability of such support are often studied in isolation without considering how HCPs interact with the wider network members and the support they provide. Although there is a range of HCPs that are involved in providing good care for patients at home, it is most frequently the district nurse caring and treating that person.^17^ Therefore, exploring the relationship dynamics between patients and district nurses may be beneficial when taking a social network approach to exploring healthcare at home. This may include how patients engage with network support when interacting with district nurses; the role of different ties and how network engagement might be in tension or complement district nursing support; how such processes and relationships co‐shape the provision of community services; and how dynamics might differ from what is already known about LTC self‐management support when self‐managing increasingly complex health conditions at home.

Furthermore, primary services like district nursing and other community nursing services such as HAH are more likely to occur in rural contexts where adapting to financial challenges has resulted in the scaling back of smaller, more remote hospitals^18^; and as such, these localities must also be considered when exploring the role of district nurses in patient social networks. The definition of “rural” varies globally with, for example, the UK describing it as areas that fall outside of settlements with more than 10,000 residents^19^; and in Australia, as all areas outside of major cities.^20^ Similarly, there is no standard definition of “rural” within healthcare^21^ but there is a consensus that the pressures and complexities of district nursing are exacerbated by rurality. This is in part because HCPs need to become generalists, healthcare services appear underfunded, operating in a context of poor infrastructure and services provided over long distances.^22^, ^23^ Furthermore, the lack of peer support paired with the diverse patient group that district nurses treat can cause stress and poor staff retention among the workforce,^23^, ^24^ all of which could impact on district nurses' ability to provide self‐management support in rural areas. There is some evidence to suggest that district nurses often live within, or near, the communities they serve,^4^ which may offer an opportunity for drawing on existing relationships, shared values, and local embeddedness to provide a motivation to overcome rural challenges. Whether this affects the way social network support is provided or whether it is qualitatively different from other urban settings, requires further exploration.

These factors contribute to the growing demands and complexity of healthcare and self‐management support in rural areas and the growing pressures on healthcare professionals, individuals, and other members of their personal communities. The pressure on community‐based services, in particular, is likely to increase because of the overwhelming demands on inpatient care, and current strategies and policies outlined in the NHS Long Term Plan,^2^ which encourage community‐based public health interventions for increasingly complex and acute needs. These require patients and their social network to take greater responsibility for the management of their health conditions, which previous research has shown is a challenging prospect for patients when confounded by reduced function caused by poor health.^11^ This qualitative metasynthesis will explore the role that district nurses can play within the wider networks of people who are currently receiving professional medical care at home, for a diverse range of conditions, while living in rural areas. It will aim to identify the formal and informal processes that shape the involvement of HCPs with the self‐management support of patients and the engagement with members of their social networks.

## 2 AIMS

The review will synthesize the available evidence on the use of district nursing services to explore:

1. The way in which district nurses develop relationships with service users to mobilize and/or become part of their personal network and what impact this has on the ability to deliver good care.
2. How rurality affects professional‐patient interactions, social network dynamics, and the ability to fulfill social, emotional, and practical needs.

## 3 METHODS

Metasynthesis offers a rigorous and systematic approach to reviewing and analyzing the literature that allows the development of novel interpretations while ensuring that the findings are reliable and transferable.^25^

### 3.1 Search strategy

The literature search was undertaken by JG in CINAHL, Medline, and PsychINFO using terms related to social networks, rurality, and community nursing, as guided by an abbreviated version of the PICO (Population, Intervention, Context/Comparison, Outcome) framework (see Table 1 for search terms). The search was completed on July 25, 2019, using the article title and abstracts only. Initial scoping searches identified limited articles that contained all three themes (ie, social networks, rurality, and community nursing), therefore, the decision was made to undertake two separate searches: the first combining “social networks” and “district nursing,” and the second searching for articles related to “rurality” and “district nursing.” A systematic hand search was conducted on the reference lists of existing literature reviews within the search results to find any other relevant articles that may have been missed by the search strategy or poorly indexed.^26^

**TABLE 1:** Search strategy showing the synonyms and Boolean phrases used to find all relevant articles for screening

S1	AB “Social Participation” OR AB “Social Inclusion” OR AB “social exclusion” OR AB “social Isolation” OR AB “Social relationship” OR AB “Social support theory” OR AB “Social support network” OR AB “Social support” OR AB “Social network”	129,777
S2	AB “ District nurs^*^” OR AB “ community nurs^*^” OR AB “Hospital at home” OR AB “hospital in the home”	7,132
S3	S1 AND S2	125
S4	AB “Rural health” OR AB “rural healthcare” OR AB “Rural^*^” OR AB “Rural nursing”	241
S5	S4 AND S2	229
S6	S3 OR S5	354

### 3.2 Article selection

**FIGURE 1:**
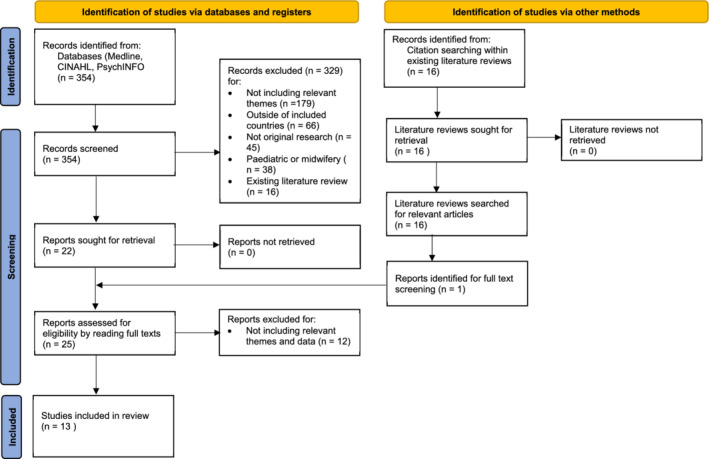
An adapted PRISMA (Preferred Reporting Items for Systematic Reviews and Meta‐Analyses) flow diagram of the article selection and screening process^28^

To be eligible for inclusion, studies had to originate from the United Kingdom (UK), Europe, United States of America (USA), Canada, Australia, and New Zealand and be published from 1st January 2009 to 1st May 2019. This was to ensure cultural consistency between the data and to ensure the synthesis was relevant to current practice. Only qualitative or mixed method studies that were written in English were included (see Table 2). Articles reporting mixed methods were included^27^ (n = 1) but only the qualitative data (quotations from district nurses' reflective accounts through semi‐structured interviews; and the interpretations made by the original authors) was extracted when reading the full texts. Social networks were defined as personal communities of individuals that provide emotional, practical, or health support, therefore, any articles relating to online networks (such as social media, often referred to as “social networks”) or telehealth approaches were excluded. Rurality was included regardless of the defining characteristic chosen by the authors of the original research (eg, population, distance to urban centers) and acknowledged during analysis. Figure 1 outlines the number of studies included and excluded at each stage of the identification and screening process. JG screened the full 354 articles found from the search at abstract level and at full text level if uncertainty remained. To ensure quality control, IV and RB each reviewed a separate 25% of the search results and the included/excluded studies were discussed until consensus was reached. Studies were excluded (n = 343) for not including relevant themes (n = 179), from outside the aforementioned westernized countries (n = 66), not including research data (eg, scoping searches or opinion) (n = 45), on pediatric care or midwifery (n = 38) or if it was an existing literature review (n = 16). Thirteen articles met the criteria for inclusion. A further article was included after hand‐searching from existing literature reviews: a total of 14 studies were, therefore, included in the final review. The articles were assessed for quality using the recognized “*Criteria for the evaluation of qualitative research*” tool for sociological research^29^ (see Table 3 for acknowledged limitations related to quality criteria of each study). Five articles focused primarily on the community healthcare professional's role, five on rurality's impact on healthcare, and three articles addressed both themes (see Table 3 for an overview of included studies).

**TABLE 2:** The inclusion and exclusion criteria used during the screening process

Inclusion criteria	Exclusion criteria
• Relevant to themes (social networks and synonyms, district nursing and synonyms, rural and synonyms)	• Does not include relevant themes
• Published since 2009	• Published before 2009
• Written in English	• Not written in English
• Qualitative study (or qualitative data of a mixed methods study)	• Quantitative study
• Originate from the UK, Europe, USA, Canada, Australia, New Zealand	• Does not originate from the UK, Europe, USA, Canada, Australia, New Zealand
	• Existing literature review

**TABLE 3:** Overview of included studies

Number, author, year, country	Study aims and objectives	Sample and context	Methodological approach	Data collection and analysis	Content supporting social networks (SN)	Limitations
1. Crotty et al,^30^ South Australia	To explore the experiences of patients with MH conditions and LTCs on their social network support.	N = 29. Users of community mental health services.	Semi‐structured interviews	Potential Participants screened by community nurse. Grounded theory	Spouses are an important member of social networks, interacting and increasing the role of formal support. However, those in this group (LTCs and MH problems) have smaller networks and often befriend DNs for their practical and emotional work. This group shows mostly transient relationships and a degree of isolation.	Convenience sampling limits rigor and single site limits transferability
2. Devik et al,^31^ Norway	To explore the views of patients on the effect long distances and poor infrastructure has on their EoL care.	Patients over 65 diagnosed with advanced cancer (n = 9) and receiving EoL care at home in rural Norway.	Narrative, semi‐structured interviews with patients. Using open ended questions	Convenience sampling. Phenomenological hermeneutic approach to analysis. Iterative approach from naïve to comprehensive interpretations	Despite being aware of worsening health outcomes patient would rather stay at home in the rural community. They feel they are able to remain a part of the community and hold some social capital and as such can draw on their relationships for support. Conversely, nursing visits restrict freedom of the patient	Authors have preconceptions as practicing nurses. Small cohort
3. Farmer and Kilpatrick,^32^ Scotland and Tasmania	Can front line HCPs stimulate changes in healthcare through entrepreneurial skills and could policy makers encourage HCPs into this role?	38 HCPs (Tasmania n = 15, Scotland = 23) from rural areas. Primary healthcare services (GPs and DNs)	Mostly face‐to‐face (n = 31) semi‐structured, “exploratory” interviews. Otherwise telephone (n = 7)	Participants recruited by advertisement, word of mouth and self‐selection at research sites. Exploratory interviews were transcribed and thematically analyzed	HCPs can built patient's social capital by identifying needs and then bridging and bonding to others. They feel obliged to become part of the community and use their own social capital to create opportunities for others. Some extreme examples where HCPs set up banks, shops, clubs for the communities. Large scale changes were mostly carried out by healthcare management and GPs while district nurses set up health clubs and health related activities.	Hard to generalize outside of the two countries. Self‐selection biases.
4. Findlay et al,^33^ Scotland	To increase the knowledge of emotional effects of living with frailty	N = 11. Semi‐structured interviews with patients at a medical day	Longitudinal qualitative study. Semi‐structured interviews	Secondary analysis of data from PhD study (n = 13). Thematic analysis	Patients wish to stay in their own home but only find contentment in doing so if they are able to connect with family and friends. Their health needs often become a barrier to maintaining relationships due to reduced mobility and district nurse visits restricting their free time.	Secondary analysis (n.b. original author on research team)
5. Gossett‐Zakrajsek et al,^34^ USA	How do older adults and HCPs experience and perceive transitions from hospital or integration back into communities?	HCPs (n = 7) from a “home health service” and patients (n = 6) recent discharged from inpatient care	Participatory Action Research. In context observation and interviews (conducted in pairs or triads)	Purposeful sampling. Field notes and interview transcriptions. Thematic analysis	Three main themes emerged on transmission to home: social support, communication, and reintegration. Informal care was highly valued by patient and HCP. Informal support allows for greater personalization, flexibility and planning of care but relies on good communication/collaboration between patient, carer, and HCP.	Observations abandoned after 6. Carer involvement unplanned for. Lack of sample diversity
6. Griffiths et al,^35^ England	How and why do DNs construct early support visits in EoL care?	DNs (n = 58) and patients (n = 10) who give or receive EoL care at home	Multiperspective. Qualitative focus groups, semi‐structured interviews, observation	Self‐selected nurses and patient Recordings and written field notes (collected by nurse researchers) transcribed and thematically analyzed	During EoL care at home, district nurses intertwined their health tasks with “having a chat.” This created an egalitarian and humanistic relationship. Nurses felt this empowered the patients and their carers to take a lead role in their treatment. Themes emerging from the data were enlightenment, explanation and education, advice and instruction.	Self‐selected participants patients likely to be most skilled and confident practitioners
7. Grunberg et al,^36^ Sweden	To use the experiences of DNs on detection and delivering mental health care to increase knowledge of good care	N = 25 DNs from Swedish community setting	Qualitative focus groups and interviews	Recruited using “snowballing” chain sampling. Same interviewer throughout. Transcriptions thematically analyzed	District nurses offered emotional support through informal dialog by trying to lift their mood and express emotions, This was important for successful integration into the SN, which gave the district nurse the ability to advocate, mobilize others, and meet emotional needs.	“Snowballing” recruitment means participants encourage only those with the same values.
8. Hunsberger et al,^21^ Canada	Considering staff and resources shortages paired with varied and complex nature of rural healthcare practices, there is need to evaluate the workforce and how to sustain/improve it.	Nurse administrators (n = 21) and Staff nurses (n = 44) from Local Health Integrated Network, Ontario. Approximately 100 miles to a large hospital	Qualitative, Semi‐structured interviews using previous research to guide themes for discussion	Recruitment using flyers and “snowball” technique. Transcripts thematically analyzed	The rural district nurse is likely to find the challenges unique to rural healthcare a stressor. These often outweigh the positives aspects and result in changing posts. The expectation to perform tasks outside of health work disgruntles staff. Demands, aging workforce and poor resources suggest rural nursing difficulties will worsen. To improve recruitment should target those from a rural background and education should have rural healthcare modules.	Self‐selection and Snowballing recruitment means nurses encourage those with shared values to participate. Increasing bias
9. Kaasalainen et al,^37^ Canada	To explore nurses' experiences of providing palliative care in rural areas with a particular focus on the impact of the physical residential setting.	District nurses (n = 21) who provide EoL care in rural communities	Qualitative exploratory techniques. Semi‐structured interviews	District nurses recruited from previous quantitative survey. Purposeful sampling. Telephone interviews thematically analyzed. Interpretations shared with participants to ensure credibility	Rural district nurses face unique physical and emotional challenges to deliver EoL care. They frequently go beyond their role for the patient. The geographical distances meant support, supplies, and patient contact time were restricted. Isolated patients made them more reliant on the district nurse and made poorer health choices of their own.	Single nursing site reduced transferability.
10. Reed et al,^38^ Australia	How do DNs successfully advocate for rural Australian EoL care goals?	DNs (n = 7) from a nursing agency in rural Victoria, Australia	Pragmatism. Written reflective accounts and follow up semi‐structured interviews.	Care agency DNs approached to take part voluntarily. Iterative analysis of reflective accounts. Semi‐structured interviews transcribed and thematically analyzed	District nurses need to become generalists in the rural community as there is little/no specialist support. The district nurse can find support and resources due to their knowledge of the community. This requires flexible relationship boundaries as they often know the patient socially. The district nurse integrates into the patient's family network and build strong rapport. This creates a reciprocal and trusting relationship that can facilitate holistic care	One practice setting limits transferability. Small sample
11. Reed et al,^27^ Australia	To create an initial understanding of how nurses practice EoL care in rural areas to provide a platform for further research that could inform practice	District nurses (n = 7) who deliver EoL care in rural areas of Australia. Wide spread areas across all states.	Sequential mixed methods. Nurses wrote reflective accounts the follow up semi‐structured interviews	Recruited district nurses purposefully selected from initial those who completed a wide spread Likert style questionnaire. Reflections used to guide semi‐structured interviews. Thematic analysis.	District nurses reported knowing the rural area so knew what resources were available. They have a good social capital so can advocate successfully. There is an issue with boundary crossing and confidentiality as the nurse often knows the patient and their family socially. They justify this by demonstrating the likely improved health outcomes. DNs have the emotional intelligence to manage this.	Self‐reporting and reflections rely on timely completion and memory. Small sample size
12. Roden et al,^39^ Australia	To explore the strategies and sustainability of the health promotion (HP) role of the rural and urban DN	10 district nurses from varying settings (rural n = 5, urban n = 5) of New South Wales, Australia	Semi‐structured Interviews following up on Likert questionnaire on the self‐efficacy and burden on HP	DNs approached to participate in quantitative study then purposeful selection for interviews. Transcriptions thematically analyzed	There is a lack of multidisciplinary support for DNs in rural areas. Their commitment to the community means the district nurse feels responsible to undertake health promotion activities and find them to be successful because they are valued members of the community. Health promotion is usually sacrificed when rural healthcare pressures build.	Only in New South Wales. Small sample interviewed.
13. Terry et al,^40^ Australia	To identify, which health and safety issues impact on the provision and quality of rural DN care What strategies to DNs adopt to overcome these?	Experienced district nurses (n = 15) from three rural care areas, Australia	Phenomenological approach. Semi‐structured interviews	Sample spread across 3 recruitment sites. Conducted interviews face to face (n = 4) or telephone (n = 11). Transcripts thematically analyzed and consensus reached.	Health and safety issues for rural DNs are primarily environmental; mainly long distances, isolation, poor infrastructure, and patient families creating difficulties. Management processes are poor so district nurses felt they had to “make do.” A strategy was to rotate caseloads to share burden of long distances to some patients and overburden of client contact. However, this negatively impacted on continuity of care.	Telephone interviews limits ability to clarify, probe, and interpret body language.
14. Wang et al,^41^ Norway	What was important to the service users of a new HaH service; to guide planning in the future	Six patients transferred to HaH service in Norway	Nine patients recruited from concurrent quantitative study comparing inpatient to HaH experience	Six patients were randomized to HaH treatment. Semi‐structured interviews were transcribed and thematically analyzed by a team of researchers	Participants discussed how they felt being treated at home compared to inpatient settings. Their social network was cited as a source of support during this time. Patients also discussed the relationship they shared with the district nurse and what work they undertook; which included some practical and emotional work alongside their healthcare role.	Small cohort, recruited from one inpatient setting limits transferability.

### 3.3 Data extraction and translation

Two data types were extracted from the articles and organized in a table that also enabled the key information of each study, such as authors, publication dates, methodology, and country of study to be easily managed (Table 3). Of the two types of data, in the first order, data included direct quotes from participants and verbatim extracts from the results chapters of each paper. Second‐order constructs (the theories developed by the researchers of the original studies) were extracted from the discussions and analyses chapters of the original articles. As is best practice when conducting a metasynthesis, in order to assess reliability, 30% of articles from each search were data extracted by members of the study team; IV and RB.^25^ The findings were discussed and consensus reached on the data that should be included, and any areas of contention throughout data extraction were discussed between the research team. From the 14 included papers, 220 first‐order quotations relevant to personal relationships, support, and relationships provided by district nurses or rural factors were included to answer the aims of this literature review. A further 83 second‐order constructs by the original authors were extracted.

Translation and reconfiguration of the data is arguably the most subjective stage of the synthesis process,^25^ and therefore, as with the other stages of this metasynthesis, findings and interpretations were discussed, revised, and elaborated within the study team.^42^ In this synthesis, in order to translate the findings into one another and develop new meaning and understanding from the included themes, a line of argument synthesis was applied. This approach allowed data from primary studies that had different contexts, and theoretical and methodological approaches to be combined.^43^ In doing so, new theories about the phenomena, the *third‐order* constructs, were developed.^25^, ^43^ This was an iterative process of repeated reading that identified recurring and juxtaposing results that could be translated into one another and identify the novel themes.^26^ Through this process the novel themes developed by the review team were: *Blurred boundaries between the types of work nurses in rural areas*; *Transactional and friend‐like nurse‐patient ties in rural localities*; *Negotiating professional responsibilities and network engagement*; and *Local embeddedness and shaping relations within local communities*. Table 4 provides an overview of the synthesis process.

**TABLE 4:** Summary of synthesis process using second and third order processes

Number, author, year, country	Translatable concept	Summary of second order interpretations by original author	Summary of third order interpretations
1. Crotty et al,^30^ South Australia	Spousal networks, the impact spouses/no spouse has on use of DN.	Spousal networks were denser and more likely to be maintained. Spouses interact with DNs and improve their significance within the SN. When there is no spouse, friends are more valuable; when there is no friendship support DNs fill friendship roles. LTCs alongside poor mental health makes maintaining networks difficult so transient SN members are frequently used.	The DN acts as a conduit to other services adding more “weak,” “transient” relationships into the SN. This usually results in a network dominated by HCPs that is unlikely to provide long‐term support and will be biomedically framed. This will likely lead to a lack of support with practical and emotional work; especially considering the mental health with long term condition cohort of this study.
2. Devik et al,^31^ Norway	Values of patients on rural healthcare and how it affects the quality of care.	Patients have to adapt to the change in lifestyle when requiring DN input in rural areas due to the interventions being delayed, interrupting schedules and routines, and being not readily available. Despite this being exacerbated by rurality patients prefer to remain here due to being a “brick” in history and place giving them increased social capital and a sense of self, security and control.	The social capital retained by aging in place means patients can retain their existing ties to help meet health, emotional and practical work. DNs are less likely to become part of the patients SN due to their visits being infrequently, on a healthcare schedule, and therefore, difficult to mobilize by the patient. Patients prefer to navigate the community to find support but targeted those with knowledge of healthcare (e.g. retired nurses).
3. Farmer and Kilpatrick,^32^ Scotland and Tasmania	HCPs outside of their healthcare role creating opportunities for patients to increase their function and social capital. In rural areas.	The DN role in a rural community is both bonding and bridging with others. Nurses have been known to be embedded in the social fabric of the community and their remit extends to lift‐giving, delivery and involvement in community facilities. They use their social capital in communities to implement change and establish entrepreneurial services (often outside of healthcare, such as establishing social clubs).	DNs use their position within the network to identify patient, SN and community needs. Especially true in rural areas where resources are limited, DNs go beyond their role and take it upon themselves to meet the needs. They also bridge and bond to other services (often out of reach to the rural patient) as a form of mobilizing others into the SN and improve the patient's social capital. Although not explicit in the data the improvement in social support and capital is likely to improve health; the DNs overarching goal.
4. Findlay et al,^33^ Scotland	Importance of a connection with family and friends when being treated at home.	Frustration and sadness at the lack of support were frequently cited, with contentment noted when friends and family were accessible. The timing of DN visits seem to restrict the ability to maintain existing relationships.	Some participants enjoyed the social aspects of the DN visits but due to timing most found them to be restricting and display ambivalence toward the service and the loneliness it brings. Ideally, individualizing services should reduce loneliness and improve associated mental and physiological health (e.g. frailty).
5. Gossett‐Zakrajsek et al,^34^ USA	The balance of formal and informal SN members during transitions from hospital to home.	Support, communication and reintegration are the three themes identified during transitions home. Successful transitions occur when there is synchronicity between formal and informal support that is planned with an appreciation for the changes occurred during time spent in hospital. There needs to be good communication between patient and informal and formal support.	A need for acute care creates changes to the patient's function and a break from their existing SN support. If there are weak ties these may disperse during inpatient stays. DNs should consider this, communicate with other SN members to overcome new shortcomings in the SN. The support provided of specialist community services at this time seems less likely to be accepted than the generalist DN support due to a stronger tie, frequency of interaction, and therefore, trust.
6. Griffiths et al,^35^ England	Relationship between DN and patient during EoL care.	DNs give information, advice, education and instruction. DNs carry out this work in a relaxed manner that empowers the patients, becomes egalitarian, and therefore, therapeutic. The physical tasks and assessment are intertwined with the social aspects of their visits; often unnoticed by the patient. The frequency of the visits also correlated to an improved self‐efficacy.	The DNs relaxed approach allows them to integrate into the patient's SN. The egalitarian relationship, not dissimilar to a friend, allows greater information sharing in both directions. The DN, once part of the SN adds new information and support, mobilize others into the SN and help with practical work that alleviates emotional stresses that impacts on their physical and mental health; thus benefiting the patient and their SN.
7. Grunberg et al,^36^ Sweden	Emotional support/work of the DN and how this helps identify needs and improve care.	DNs use informal dialog to lift patients' mood and facilitate open discussion that helps identify further needs. This was deemed important to allow an integration into the SN meaning the DN had the role to mobilize others, advocate and meet their emotional needs. These skills are intrinsic with the DN often unaware they are detecting mental health needs. However, time and resources appear a barrier.	Despite the lack of resources DNs can utilize their relaxed approach, intertwined with planned visits, to provide emotional work; either by improving mood or identifying mental health concerns. Their role becomes similar to a friend by using general conversation and joking. They can then integrate into the SN to meet emotional needs and to identify the correct people already in the SN to mobilize where needed.
8. Hunsberger et al,^21^ Canada	Values of DNs on rural healthcare and how it affects care and themselves.	Rural healthcare can be rewarding and stressful. Often the initial attraction of open countryside become the stressor due to isolated practice. Acuity in these areas is increasing and experienced nurses are approaching retirement increasing demands on the services. More recently, only those already embedded in rural life appear to choose to work there. Urban policy and decision making frustrates rural healthcare, and therefore, rural specific training is needed.	The attraction of rural nursing is to help a community one is already embedded into. The shared community values helps integration into an SN to mobilize others. Willing DNs should nurture the attachment to the community to create stronger SN ties. However, the challenges of rural healthcare seem to outweigh the positives making recruitment a challenge. The blurring of boundaries between community member and DN cause confidentiality concerns and a burden on the DN who may be contacted out of hours or in public spaces.
9. Kaasalainen et al,^37^ Canada	Physical and emotional challenges of EoL care in rural areas.	Rural nurses experience a unique challenge when delivering care to their patients; physically and emotionally. DNs have to overcome these challenges to support the patients with their increased risk of isolation and associated poorer health choices. Nurses often demonstrate extreme measures to go above and beyond for their patients.	Lack of resources force DNs to become generalists but this increases their frequency of interaction with the patient. They negotiate with the SN and community on behalf of the patient. They advocate and mobilize others into the network where possible, which could burden the DN and confidentiality issues may arise when discussing the patient with the community. DNs nurture their tie with the SN by completing practical work like stoking the fire.
10. Reed et al,^38^ Australia	Rural DNs working and living in the same area and how this influences their role.	Successful EoL care requires DNs to be committed to the emotional work involved. Resonating with other studies, the knowledge of the people and resources available in the rural community was noted as valuable in advocating to the appropriate people. The DN needs to have a flexible relationship boundary with the patient create a reciprocal relationship for confident advocacy and emotional support.	EoL care increases the importance for DNs to become part of the SN of the patient and their family and should be encouraged. Increased tie strength and involvement in emotional work creates a reciprocal relationship and shared values that are conducive to good care. There is a risk of confidentiality breaches when advocating to other members of the rural community (e.g. priests). There is also a risk of emotional burden for the DN as they are likely to be unsupported in the rural setting.
11. Reed et al,^27^ Australia	How local knowledge influences the way DNs care in rural areas.	DNs consider the values of patient and family to personalize care. Knowing the available resources in the rural area helps gather support for the patient. DNs have strong community relationships that empower them to advocate successfully. DNs possess the emotional intelligence to manage a personal and professional relationship and can justify it because of the likelihood of improved outcomes.	DNs face a challenge to meet patient's needs by nurturing a strong SN tie that could overburden them emotionally at EoL. If this is achieved they can successfully mobilize other members of the community and healthcare services. The challenges of burden, confidentiality and emotional distress are overcome by the DNs emotional intelligence.
12. Roden et al,^39^ Australia	DNs use their social capital for health promotion (HP). The pressures on rural DNs make this hard to sustain.	There is a lack of support and competing priorities for a rural DN. However, DNs had a more positive and committed outlook on HP, possibly because they knew and felt responsible for the community they served. Patients were more likely to follow the HP advice due to the respect they had for the DN. HP was often sacrificed when rural challenges (workforce, infrastructure, resources) limited their availability to patients causing stress and disengagement by the DN.	Effective HP requires committed DNs to be embedded into the community. This increases the tie strength between patient and DN and the patient is then more likely to follow advice. Relationships are mutual, open and conducive to honest reporting of health behaviors. Rurality acts as a facilitator to shared values, community engagement, and therefore, stronger tie but also restricts the time and resources available to deliver HP effectively. DNs may neglect HP as a result of rurality to treat the patient's primary health need.
13. Terry et al,^40^ Australia	The health and safety (HS) issues of rural healthcare and how it affects quality of care.	HS issues are complex and largely environmental. These include isolation, long distances, and poor infrastructure. DNs cite “making do” and developing skills to overcome the HS issues. This includes rotating staff and dividing workload. Lack of funding, support, supervision, and specific training exacerbate the problem and result in poor staff retention.	The needs of the DN and patient cannot be met in parallel. The HS issues in rural communities result in DNs create physical and emotion distress among DNs. The coping strategies used makes them less available to the patient, poor continuity of care, and therefore, a weaker tie is developed within the SN; encompassed by patients “making do with who turns up” and “not speaking to them in the same way.”
14. Wang et al,^41^ Norway	Comparing experiences of being treated for acute illness at home or as an inpatient	A surprising finding was that participants at home felt safe despite the limited time with an HCP. This accredited to the ability to communicate via telephone at anytime and also the personalized care plans. Being treated in their own home improved the relevance of the information and advice given; patients could relate it to their everyday life, and therefore, compliance increased. Nurses also had more time to spend 1‐to‐1.	Aging in place/being ill in place allows information/health promotion to be more relevant to the patient and their SN, and therefore, accepted and implemented. They know “what‐is‐What” will increase long term self and collective efficacy. The acceptance is also due to the increased strength of the tie that is likely to be built between the nurse, patient, and their family. This is because they are reliable, attentive, 1‐to‐1, and have the time to spend with the patient.

## 4 FINDINGS

### 4.1 The development of both transactional and friend‐like nurse‐patient ties in rural localities

Rural settings impact on the relationships between nurses and patients in several ways, and result in two key types of relationship styles with service users. The first are those that are transactional in nature, and develop as the result of relatively infrequent, discontinuous, and unreliable interactions between nurses and patients in community contexts.^31^ This is often due to nurses serving patients in isolated areas with poorly developed infrastructures and phone networks, long distances and travel times between patients, and high levels of staff turnover and rotation (especially where long distances need to be shared).^37^, ^39^, ^40^ Within such contexts, patients have to “*take whoever, whether you like them or not*”^33^ meaning the building of trusting and therapeutic relationships between patients and nurses might become difficult.^36^, ^40^ Patients cite that they “*never feel like discussing things with them [district nurses they see less frequently] in the same way*”,^33^ which contributes to relations in rural areas, which feel transactional, fleeting, and impersonal, although not necessarily ineffective.^41^

However, the 1‐to‐1 contact in community settings and the interactional confidence that patients have due to being in their own homes where they “*know what is what*”,^31^ also opens possibilities for development of close, highly personalized relationships between patients and nurses, which are valued by the patient^33^, ^38^:

We have a good connection. It means a lot to me. She is more than a nurse…she is a person.^31^

Being open to the development of such “*comfortable*”^38^ relations with patients fits with the perceptions of nurses of their professional role and they see it as an achievable aim and an effective way of supporting patients.^21^, ^27^ To accomplish this, nurses may adopt certain interactional styles. For example, a “*relaxed conversation style*”^35^ and make themselves personally accessible to the patient^21^ by, for example, giving out their personal number;

your number is in the book, or you give them your personalnumber.^27^

### 4.2 Engagement of the wider network in the delivery of good care

Where nurses develop close relationships with the patient, there is evidence to suggest that the nurse may be (or become) part of the social network, as well as interact with other individuals within the wider social network. In this way, they often utilize interactions with patients as an opportunity to identify emotional and practical needs being unmet by the rest of the patient's social network,^27^, ^32^, ^34^, ^38^, ^39^, ^41^ and commonly feeling obliged to offer support in these areas.^32^, ^34^ This support may include practical tasks such as stoking the fire, “Training the dog,”^27^ organizing or providing transport,^32^ organizing financial support in the form of “getting benefits,”^32^, ^35^ and providing emotional support by spending time talking and discussing personal concerns.^35^, ^36^ The rationale for undertaking practical roles might be in order to reduce negative events such as falls when less able patients attempt to do practical work independently; or even prevent self‐neglect if patients cannot cook and wash clothing.^21^, ^38^ The emotional work undertaken by nurses during health visits may be used as a “*lever*” for further assessment,^35^ which not only reduces negative effects associated with loneliness, isolation, and poor mental health, but also acts as a technique for identifying health needs.^35^, ^36^ For example, district nurses would “*just, you know, chat about things in general…like a social visit…and sometimes by just doing that, little problems will come out*”.^35^

When district nurses live and work in the same rural locality, there are often pre‐existing relationships with the patient and/or other social network members.^27^, ^33^ For example, one nurse said she was able to help a man to “*die at home with his three teenage sons – one of which I employed locally*”.^27^ This is beneficial as it helps to create an egalitarian relationship that is based on shared norms and values.^21^, ^27^ Moreover, the nurse may be well placed not only to successfully identify potential social networks of support^37^ but also have the increased social capital within the community to enable its successful mobilization.^27^, ^32^, ^38^ The quote that “*People don't say no to a health care professional as readily*”^32^ epitomizes this increased social capital and nurses are seen as the “*quarterback*” of the community^37^; mobilizing other professionals and healthcare services.^30^, ^32^, ^35^, ^36^, ^41^ Nurses “*Bridge or bond [patients to others]*”^32^ such as churches, clubs, or charities^32^, ^36^ but also, in some cases, proactively create new social networks of support by establishing their own clubs, community projects, or shops that offer an opportunity for interaction with others in the community.^32^

### 4.3 Blurring of professional boundaries in close community relationships

However, the development of complex nurse‐patient relationships may result in some degree of crossing the boundary of one's professional role in order to fulfill key nursing responsibilities, especially in rural areas^27^, ^33^, ^35^, ^36^, ^38^, ^40^, ^41^:

individuals that carry out a formal service begin to undertake informal support roles.^30^

Nurses reported experiencing the pressure of expectations from patients to act as a substitute for the absent support from family, friends, and peers^33^; stating they “*get calls at home – A lot of calls!*”.^37^ Similarly, researchers highlighted that the familiarity patients had with nurses meant they found their “*privacy was invaded*”^37^ when they were “*consulted about health issues in grocery stores or at sports events*”.^21^ Such patient expectations are likely to be unrealistic given that rural factors outlined above restrict the time available to nurses to offer substantial emotional and practical support.^37^, ^38^, ^40^ This may leave nurses with difficult choices to make between disappointing raised patient expectations, fulfilling responsibilities to other patients, and the need to prioritize illness over all other types of work, such as domestic tasks or food shopping.^39^ Thus, close relationships between nurses and patients may be difficult to negotiate and manage,^21^ adding substantial amount of relational work to the nurse workload, and raising issues of overburden or “burnout,” confidentiality, and meeting professional and legal responsibilities and standards.^27^, ^31^

Moreover, in rural community settings where social isolation can be common^37^, ^40^ some patients may act proactively use nurse visits as an opportunity for social contact,^36^ and in the absence of network support, patients may actively seek district nurses to provide emotional and other types of support^30^, ^31^:

I see her if I come in here to say hello…I'm not actually allowed to consult with her because I'm not classed as homeless.^30^

However, when district nurses practice “*generosity exceeding what can be expected*”^31^ relationships with patients strengthen and district nurses become more forthcoming with offering additional support and may come to be “*perceived by patients as a friend*”.^38^ It may also lead to reshaping existing relations between patients and members of their wider network. Relying on professionals for emotional and practical support can cause any available, existing support to dissipate leaving the patient vulnerable if formal support is discontinued,^33^ while also putting additional pressure on nurses to further extending the depth and range of support they provide.

### 4.4 Issues accessing and navigating formal and informal support in the context of diminishing resources in rural areas

While building close nurse‐patient relationships may sometimes be associated with higher personal job satisfaction,^21^, ^38^ the need to deal with complexity that such relations introduces associated negative experiences^27^ maybe less acceptable to newly qualified nurses who “*may not be comfortable with all the different things [emotional and practical support] they had to do*,” according to their more experienced peers.^21^ As with the community as a whole, smaller rural district nursing teams experience an increased sense of shared values and team spirit among themselves^38^ and are able to create an “*extended family environment*”^21^ but there is a relative lack of specialist support available; which not only means nurses practice as generalists but also that it restricts the services available that can be mobilized to support the patient.^21^, ^27^, ^37^, ^38^, ^39^ Furthermore, the aging workforce in rural areas means that recruitment from outside the local area is increasingly common. This reduces the embeddedness and shared values of the nurse in “*both a geographical and social sense*,”^31^ limits the knowledge the nurse has of the community, and therefore, the influence they have to mobilize other forms of support.^21^ Consequently, despite aiming to increase the social network of support through advocacy and mobilization of others, in an attempt to improve efficiency or through an unawareness of the local community dynamics, district nurses unintentionally limit the patient's social capital if they are unable to participate in their usual social network interactions because of time‐conflicting health interventions; arranged at a time to suit the professional.^31^, ^33^ For example, patients feel “*your life's not your own*”^33^ and that “*much time is spent waiting…there might be other things you would rather like to do*”.^31^ Patients with LTC and poor mental health have a more frequent use of paid and formal care use, and are, therefore, particularly vulnerable to this.^30^

## 5 DISCUSSION

This qualitative metasynthesis found that HCPs who work in rural areas are involved in wide ranging support for patients. This work goes beyond their professional specialty and experience and may include providing social and emotional support, help with daily tasks, and building links to local communities. Our findings indicate that taking on such a complex role is needed in order to provide effective and safe care for people living in rural areas. This review has found two dominant models in terms of how this is currently done in terms of developing relationships with patients and engaging with their wider networks of support: one model where nurse‐patient relations are kept at an arms‐length and another where nurses develop close relations with patients, which resemble friendships, with links extending to their wider networks, including families, friends, and the localities where they live. These findings indicate that neither of these models is optimal for delivering patient‐centered care in the community. In the case of the former, this is in part due to lack of understanding of the patient context, resources, and structure of support, with minimal or no knowledge and engagement of the wider social network members, and thus with likely negative implications for patient care and support tailored to individual needs. In the case of the latter, this is due to building expectations among patients and their network members that nurses might be able to address multiple gaps in the provision of health and social support arising from structural inequalities and the structure of people's networks. However, such relations are unrealistic and unsustainable over the longer term due to the risk of nurses becoming overburdened and because changes to healthcare service staff and provisions might make the nurse unreliable to patients; especially considering the uncertain finance, probable increase of complex community care and policy changes affecting healthcare, all of which reduces how effectively the nurse can deliver additional support.

Furthermore, as with other studies, this qualitative metasynthesis suggests that nurses developing an understanding of, and involvement with, patient's social networks does not automatically translate into positive outcomes for patients.^13^, ^44^ For example, such close ties can have negative impact on the wider network of support by restricting engagement with existing network members and the building of new links. Therefore, our findings have suggested that nurse‐patient relations in rural areas work best where nurses are seen as trusted acquaintances with a broad understanding of the social and emotional needs of patients and the financial and relational resources accessible to them. Such relationships are currently ad hoc, but they might develop, and become most effective in localities that nurses are familiar with and have greater social capital within the community because they live in the area or because they have been professionally involved with it over a long period. This may be because in such circumstances nurses are more likely to be familiar to patients, their family members and the wider local community either directly or indirectly through personal and professional reputation and support. These weak ties with patients paired with an understanding of local and individual structures of support, can allow nurses to help patients find, access, and mobilize other network members in a way that is acceptable to them^45^, ^46^ but also make them aware of new relationships and support that might be available, thus increasing the diversity of support and information.^11^, ^16^ Such relations with patients are likely to be sustainable over the longer term as, they are contextually sensitive, but also compared to strong ties, require lower levels of relational work (eg, in negotiating acceptable engagement with other network members) and thus reduce the risk of burnout of nurses. Adapting the role of DN would allow them to improve collaborative work with people's informal network members while also delivering care that is better tailored to patient needs and context.

### 5.1 Implications for practice

Engagement with patient's social networks is likely to add value for patients living in rural areas and for community‐based nursing teams. However, expectations for developing close relations with patients as a part of the nursing role should be seen as unrealistic considering the tensions between the growing complexity, demand, and availability of services; but also due to additional tensions that such relations, and relational work (the interpersonal efforts that district nurses will invest in order to develop relationships between themselves and the patient,^47^ are likely to create. Developing weak ties of trustworthiness and familiarity with patients is consistent with the nursing role and is likely to help with providing effective patient care. In developing such relations, district nurses could focus on using health interactions to engage in conversations about family, friends, and peers; and what they do or do not do to support the patient. Such knowledge, together with awareness of local resources and informal support, can allow nurses to help patients shape relations with their network members, access, and negotiate relations within the community and healthcare services, mobilizing other sources of support that can diversify the patient's existing network. This will add sustainability to the support; improve patient outcomes associated with improved health, practical, and emotional support; and reduce the potential burden of responsibility on the healthcare service and professional. However, such relations between patient and nurses are currently only developed ad hoc. Making them sustainable in the context of increasing acuity and demand is likely to require putting in place support for professional development, and building resources and infrastructure enabling links between relevant professional and community resources and support (eg, health trainers, social prescribers, befriending services).

## 6 CONCLUSIONS

This review used the systematic approach of a qualitative metasynthesis in order to gain insights into the effect of rurality on district nurse‐patient relationships, where existing data had previously focused on the two themes in isolation. The focus of this review was to combine and address how the two factors influence, compliment, or conflict with one another; and develop further understanding of what approaches patients and professionals should adopt in these contexts. Findings demonstrated that HCPs in rural areas cross boundaries, first, with the work they carry out, and second, from a professional relationships one similar to friendship. There was also evidence of local embeddedness and nurses negotiating with the community in order to find support on the patient's behalf. The discussion demonstrated that nurse‐patient‐social network relationships can be unsustainable if they are burdensome or disruptive to existing social networks. Developing weak ties of familiarity with patients and building awareness of, and connection to, local structures of support is likely to offer a promising avenue for developing community‐based nursing support that is sustainable and tailored to patient needs. In this regard, this review contributes to the understanding of the key role that weak ties play in people's networks by exploring such ties in a different context and focused on healthcare professionals, but further research is needed, across varying community nursing services, in order to develop a clear understanding of the dynamics of such a role and relationships and the necessary conditions and resources that might be needed for their embeddedness into practice.

## 7 LIMITATIONS

This review included only qualitative studies. Although this method fills gaps in understanding and underlying mechanisms left by quantitative studies, qualitative synthesizes cannot include the number of studies of a quantitative synthesis. Furthermore, as a review of a previously unexplored areas of rural healthcare paper, the outcomes identified are theoretical and may require empirical investigation to confirm.

## FUNDING INFORMATION

The university of Southampton and the Dorset County Hospital have jointly funded the PhD programme of JG and therefore this study. Open access funding provided by the University of Southampton.

## TRANSPARENCY STATEMENT

Jack Gillham affirms that this manuscript is an honest, accurate, and transparent account of the study being reported; that no important aspects of the study have been omitted; and that any discrepancies from the study as planned (and, if relevant, registered) have been explained.

## CONFLICT OF INTEREST

The authors declare that they have no conflicting interests.

## AUTHOR CONTRIBUTIONS

Conceptualisation: Jack Gillham, Ivaylo Vassilev, Rebecca Band

Data Curation: Jack Gillham, Ivaylo Vassilev, Rebecca Band

Methodology: Jack Gillham, Ivaylo Vassilev, Rebecca Band

Writing: First Draft Preparation: Jack Gillham

Writing: Review and Editing: Jack Gillham, Ivaylo Vassilev, Rebecca Band

Supervision: Ivaylo Vassilev, Rebecca Band.

All authors have read and approved the final version of the manuscript.

Jack Gillham had full access to all of the data in this study and takes complete responsibility for the integrity of the data and the accuracy of the data analysis.

## ETHICAL STATEMENT

This is a qualitative metasynthesis of existing papers, all of which had ethical approval.

## Data Availability

The authors confirm that the data supporting the findings of this study are available within the article and its supplementary materials.

## References

[1] Taylor D, Bury M. Chronic illness, expert patients and care transition. Sociol Health Illn. 2007;29:27‐45. 17286704 10.1111/j.1467-9566.2007.00516.x

[2] NHS England . 2019. *NHS Long Term Plan* [Online]. https://www.longtermplan.nhs.uk/wp-content/uploads/2019/08/nhs-long-term-plan-version-1.2.pdf. Accessed November 20, 2019.

[3] Anandaciva S. Financial Debt and Loans in the NHS. London: The Kings Fund; 2020.

[4] Barrett A, Terry DR, Lê Q, Hoang H. Factors influencing community nursing roles and health service provision in rural areas: a review of literature. Contemp Nurse. 2016;52:119‐135. 27264878 10.1080/10376178.2016.1198234

[5] Kennedy A, Vassilev I, James E, Rogers A. Implementing a social network intervention designed to enhance and diversify support for people with long‐term conditions. A qualitative study. Implement Sci. 2015;11:1‐15. 10.1186/s13012-016-0384-8 PMC4772323 26926837

[6] Pearson M, Hunt H, Cooper C, Shepperd S, Pawson R, Anderson R. 2013. The effective and cost‐effective use of intermediate, step‐down, hospital at home and other forms of community care as an alternative to acute inpatient care: a realist review. London: NIHR Health Services and Delivery Research Programme.

[7] Kennedy A, Reeves D, Bower P, et al. The effectiveness and cost effectiveness of a national lay‐led self care support programme for patients with long‐term conditions: a pragmatic randomised controlled trial. J Epidemiol Community Health. 2007;61:254‐261. 17325405 10.1136/jech.2006.053538 PMC2652924

[8] Vassilev I, Rogers A, Sanders C, et al. Social networks, social capital and chronic illness self‐management: a realist review. Chronic Illn. 2011;7:60‐86. 20921033 10.1177/1742395310383338

[9] Vassilev I, Rogers A, Blickem C, et al. Social networks, and the ‘work’ and work force of chronic illness self‐management: a survey analysis of personal communities. PlosONE. 2013;8(4):e59723. 10.1371/journal.pone.0059723 PMC3615067 23565162

[10] Reeves D, Blickem C, Vassilev I, et al. The contribution of social networks to the health and self‐management of patients with long‐term conditions: a longitudinal study. PLoS One. 2014;9:e98340. 24887107 10.1371/journal.pone.0098340 PMC4041782

[11] Vassilev I, Rogers A, Kennedy A, Koetsenruijter J. The influence of social networks on self‐management support: a metasynthesis. BMC Public Health. 2014;14:719. 25023948 10.1186/1471-2458-14-719 PMC4223639

[12] Walker S, Kennedy A, Vassilev I, Rogers A. How do people with long‐term mental health problems negotiate relationships with network members at times of crisis?. Health Expect. 2018;21(1):336–346. 29024284 10.1111/hex.12620 PMC5750694

[13] Rogers A, Vassilev I, Kennedy A, Blickem C, Reeves D, Brooks H. Why less may be more?: A mixed methods study of the work and relatedness of ‘weak’ ties in supporting long term condition self‐ management. Implement Sci. 2014;9(1):19–29. 24524253 10.1186/1748-5908-9-19 PMC3932842

[14] Christakis N, Fowler J. The spread of obesity in a large social network over 32 years. N Engl J Med. 2007;357:370‐379. 17652652 10.1056/NEJMsa066082

[15] Christakis N, Fowler J. Connected. London: Haper Press; 2009.

[16] Pescosolido BA. Of pride and prejudice: the role of sociology and social networks in integrating the health sciences. J Health Soc Behav. 2006;47:189‐208. 17066772 10.1177/002214650604700301

[17] Aldridge‐Bent S. Community and public health nursing, (Vol. 5). Oxford: Wiley Blackwell; 2014.

[18] Rechel B, Džakula A, Duran A, et al. Hospitals in rural or remote areas: An exploratory review of policies in 8 high‐income countries. Health Policy. 2016;120:758‐769. 27312144 10.1016/j.healthpol.2016.05.011

[19] Department for Environment, F. A. R. A . Defining rural areas using the rural urban classification. London: Crown Copyright; 2017.

[20] Australian Institute for Health and Welfare . Rural and Remote Australians. Australian Institute for Health and Welfare: New South wales; 2019.

[21] Hunsberger M, Baumann A, Blythe J, Crea M. Sustaining the rural workforce: nursing perspectives on worklife challenges. J Rural Health. 2009;25:17‐25. 19166557 10.1111/j.1748-0361.2009.00194.x

[22] Roberts D, Hibberd P, Lewis CA, Turley J. The unique contribution of community clinical nurse specialists in rural Wales. Br J Community Nurs. 2014;19:601‐607. 25475675 10.12968/bjcn.2014.19.12.601

[23] Robinson C, Pesut B, Bottorff J, Mowry A, Broughton S, Fyles G. Rural palliative care: a comprehensive review. J Palliat Med. 2009;12:253‐258. 10.1089/jpm.2008.0228 19216703

[24] Daniels ZM, Vanleit BJ, Skipper BJ, Sanders ML, Rhyne RL. Factors in recruiting and retaining health professionals for rural practice. J Rural Health. 2007;23:62‐71. 17300480 10.1111/j.1748-0361.2006.00069.x

[25] Lachal J, Revah‐Levy A, Orri M, Moro MR. Metasynthesis: an original method to synthesize qualitative literature in psychiatry. Front Psych. 2017;8:269‐278. 10.3389/fpsyt.2017.00269 PMC5716974 29249996

[26] Noblit GW, Hare RD. Meta‐Ethnography: Synthesizing Qualitative Studies. London: Sage; 1988.

[27] Reed F, Fitzgerald L, Bish MR. Advocating for end‐of‐life choice at home: a survey of rural Australian nurses. Rural Remote Health. 2018;18:4322‐4322. 30125509 10.22605/RRH4322

[28] Page MJ, McKenzie JE, Bossuyt PM, et al. The PRISMA 2020 statement: an updated guideline for reporting systematic reviews. BMJ. 2021;372:n71. 33782057 10.1136/bmj.n71 PMC8005924

[29] Blaxter M. Criteria for the evaluation of qualitative research papers. Med Soc News. 1996;22(1):68–71.

[30] Crotty MM, Henderson J, Ward PR, et al. Analysis of social networks supporting the self‐management of type 2 diabetes for people with mental illness. BMC Health Serv Res. 2015;15:257‐257. 26138825 10.1186/s12913-015-0897-x PMC4490681

[31] Devik SA, Hellzen O, Enmarker I. “Picking up the pieces”—Meanings of receiving home nursing care when being old and living with advanced cancer in a rural area. Int J Qual Stud Health Well‐Being. 2015;10:28382. 26362533 10.3402/qhw.v10.28382 PMC4567585

[32] Farmer J, Kilpatrick S. Are rural health professionals also social entrepreneurs? Soc Sci Med. 2009;69:1651‐1658. 19783086 10.1016/j.socscimed.2009.09.003

[33] Findlay C, Lloyd A, Finucane AM. Experience of emotion in frail older people towards the end of life: a secondary data analysis. Br J Community Nurs. 2017;22:586‐592. 29189057 10.12968/bjcn.2017.22.12.586

[34] Gossett Zakrajsek A, Schuster E, Guenther D, Lorenz K. Exploring older adult care transitions from hospital to home: a participatory action research project. Phys Occup Ther Geriatr. 2013;31:328‐344.

[35] Griffiths J, Ewing G, Rogers M. Early support visits by district nurses to cancer patients at home: a multi‐perspective qualitative study. Palliat Med. 2013;27:349‐357. 22801979 10.1177/0269216312451949

[36] Grundberg Å, Hansson A, Hillerås P, Religa D. District nurses' perspectives on detecting mental health problems and promoting mental health among community‐dwelling seniors with multimorbidity. J Clin Nurs. 2016;25:2590‐2599. 27273609 10.1111/jocn.13302

[37] Kaasalainen S, Brazil K, Williams A, et al. Nurses' experiences providing palliative care to individuals living in rural communities: aspects of the physical residential setting. Rural Remote Health. 2014;14:1‐13. 24965671

[38] Reed FM, Fitzgerald L, Bish MR. Rural district nursing experiences of successful advocacy for person‐centered end‐of‐life choice. J Holist Nurs. 2016;35(2):151‐164. 27149994 10.1177/0898010116646643

[39] Roden J, Jarvis L, Campbell‐Crofts S, Whitehead D. Australian rural, remote and urban community nurses' health promotion role and function. Health Promot Int. 2016;31:704‐714. 25838551 10.1093/heapro/dav018

[40] Terry D, Lê Q, Nguyen U, Hoang H. Workplace health and safety issues among community nurses: a study regarding the impact on providing care to rural consumers. BMJ Open. 2015;5:e008306. 10.1136/bmjopen-2015-008306 PMC4538262 26270947

[41] Wang Y, Haugen T, Steihaug S, Werner A. Patients with acute exacerbation of chronic obstructive pulmonary disease feel safe when treated at home: a qualitative study. BMC Pulm Med. 2012;12:45‐45. 22920051 10.1186/1471-2466-12-45 PMC3517315

[42] Barry CA, Britten N, Barber N, Bradley C, Stevenson F. Using reflexivity to optimize teamwork in qualitative research. Qual Health Res. 1999;9:26‐44. 10558357 10.1177/104973299129121677

[43] Atkins S, Lewin S, Smith H, Engel M, Fretheim A, Volmink J. Conducting a meta‐ethnography of qualitative literature: Lessons learnt. BMC Med Res Methodol. 2008;8:21. 18416812 10.1186/1471-2288-8-21 PMC2374791

[44] Lucas S. The missing link: district nurses as social connection for older people with type 2 diabetes mellitus. Br J Community Nurs. 2013;18:388‐397. 24225474 10.12968/bjcn.2013.18.8.388

[45] Band R, James E, Culliford D, et al. Development of a measure of collective efficacy within personal networks: a complement to self‐efficacy in self‐management support? Patient Educ Couns. 2019;102:1389‐1396. 30905493 10.1016/j.pec.2019.02.026

[46] Vassilev I, Band R, Kennedy A, James E, Rogers A. The role of collective efficacy in long‐term condition management: A metasynthesis. Health Soc Care Community. 2019;27:e588‐e603. 31231928 10.1111/hsc.12779 PMC6852408

[47] Locher MA, Watts RJ. Relational Work and Impoliteness: Negotiating Norms of Linguistic Behaviour. Berlin: Mouton de Gruyter; 2008.

